# Impact of acute and chronic hypoxia on the heme oxygenase/carbon monoxide pathway in naked mole-rats (*Heterocephalus glaber*)

**DOI:** 10.1242/jeb.250502

**Published:** 2025-12-12

**Authors:** Kristi M. Kezar, Liam Eaton, Karen L. Kadamani, Mohammad Ojaghi, Leo E. Otterbein, Matthew E. Pamenter, Michael S. Tift

**Affiliations:** ^1^ Department of Biology and Marine Biology, University of North Carolina Wilmington, Wilmington, NC 28403, USA; ^2^ Department of Biology, University of Ottawa, Ottawa, ON, Canada K1N 9A7; ^3^ Ottawa Institute of Systems Biology, University of Ottawa, Ottawa, ON, Canada K1H 8M5; ^4^ Division of Gastroenterology, Hepatology and Transplantation, Beth Israel Deaconess Medical Center, Center for Life Sciences, Harvard Medical School, Boston, MA 02215, USA

**Keywords:** Carbon monoxide, Heme oxygenase, Hypoxia tolerance, Naked mole-rat, Carboxyhemoglobin

## Abstract

Heme oxygenase (HO) enzymes are responsible for the degradation of free heme and producing endogenous carbon monoxide (CO). Research has suggested that the HO–CO pathway imparts protective effects to hypoxic tissues. The objective of this study was to investigate the effects of acute (4 h or 24 h at 7% O_2_) and chronic (7 days at 11% O_2_) hypoxia on the HO–CO pathway in the hypoxia-tolerant naked mole-rat. Specifically, we measured CO concentrations in nine organs and blood, as well as HO activity in all organs of animals exposed to normoxia (21% O2), acute hypoxia or chronic hypoxia. Hypoxia did not impact CO concentration or HO activity in most tissues, with the exception of the brain (decreased [CO] after 24 h and 7 days), heart (increased HO activity after 4 h), and intestine (increased [CO] after 24 h and 7 days but decreased HO activity after 24 h). Relative to normoxic controls, hemoglobin concentrations increased 10–12% in animals exposed to acute hypoxia but were unchanged following chronic hypoxia. In naked mole-rats exposed to normoxia or hypoxia, CO concentrations were higher in blood, lung and spleen, and HO activity was higher in the kidney and lung when compared with tissues from mice exposed to normoxia. However, splenic HO activity was higher in mice exposed to normoxia when compared with spleen from naked mole-rats in all treatment conditions. Unlike non-hypoxia tolerant species, chronic hypoxia did not suppress the HO–CO pathway in naked mole-rats, highlighting the importance of this pathway in hypoxia physiology.

## Teaser

**Highlighted Article:** Variability in heme oxygenase activity and tissue carbon monoxide content in naked mole rats exposed to acute and chronic hypoxia.

## INTRODUCTION

Reduced or fluctuating environmental oxygen (O_2_) is a common occurrence in a multitude of diverse habitats in both terrestrial and aquatic environments (Bickler and Buck, 2007; Buck and Pamenter, 2006; Dzal et al., 2015). Environmental hypoxia is considered to be a critical driver of evolutionary adaptation, resulting in elegant and often novel physiological mechanisms that contribute to systemic hypoxia tolerance (Heinrich and Tift, 2023; Pamenter et al., 2020). It has been speculated that one such adaptive pathway that supports hypoxia tolerance may be the heme degrading enzyme, heme oxygenase (HO), which generates carbon monoxide (CO) (Carraway et al., 2000; Fabries et al., 2022; Herrera et al., 2008; Lewis et al., 2015; Prabhakar et al., 2018; Tift et al., 2020). HO enzymes are highly conserved proteins found ubiquitously in animals, plants and bacteria, and are the rate-limiting enzymes in the catabolism of heme into biliverdin, which is subsequently degraded into bilirubin by biliverdin reductase (Li and Stocker, 2009; Tenhunen et al., 1968; Wilks, 2002). HO enzymes require NADPH and O_2_ to catabolize free heme (a potent free radical) in tissues of organisms, releasing equimolar concentrations of CO, ferrous iron and biliverdin (Tenhunen et al., 1968). Clearance of free heme makes HO enzymes potent antioxidants, although there are several studies that have now shown that HO enzymes and byproducts (e.g. CO, biliverdin, bilirubin) have other cytoprotective effects as well (Bauer et al., 2025; Ryter, 2020; Siracusa et al., 2021). The CO that is released in animals will typically either bind to hemoglobin (Hb), forming carboxyhemoglobin (COHb) or enter extravascular tissue, where it likely binds to other heme proteins and exits the body primarily via respiration (Coburn et al., 1963; Tenhunen et al., 1968; Wu and Wang, 2005). There are two known isomers of heme oxygenase. In basal conditions, HO-1 is expressed in low to undetectable levels throughout most tissues (except in spleen, where erythrocytes are processed), whereas in hypoxia, HO-1 is upregulated (Maines, 1997; Ryter et al., 2006). In contrast, HO-2 is generally believed to be constitutively expressed and for that reason has received considerably less research attention than HO-1. However, it is known that hypoxia decreases HO-2 gene and/or protein expression in several human cell lines, whereas HO-2 protein levels decrease in mouse liver after 7 days of hypoxia and increase in the heart after 28 days of hypoxia, indicating that expression of this protein is not as static as previously suggested (Han et al., 2005; He et al., 2010; Zhang et al., 2006).

Some species and populations express modifications to the HO–CO pathway that support systemic hypoxia-tolerance. For example, *HMOX2* is under positive selection in several high altitude Tibetan human populations. This evolutionary pressure may have resulted in a gain-of-function mutation that contributes to the regulation of lower Hb concentrations and maintenance of a blunted erythropoietic response to hypoxia, thereby preventing polycythemia due to chronic mountain sickness in these populations (Simonson et al., 2010; Yang et al., 2016). In addition, when neonatal llamas (*Lama glama*; high-altitude adapted species) and sheep (*Ovis aries*; lowland species) underwent gestation and delivery at high altitude (3600 m), only the sheep developed pulmonary arterial hypertension (Herrera et al., 2008). When compared with animals that underwent gestation and delivery at low altitude, only the llamas exhibited elevated pulmonary HO-1 protein levels and CO production (Herrera et al., 2008). It was concluded that upregulation of the HO–CO pathway in the neonatal llamas helped prevent hypoxia-induced vascular remodeling and/or inducing pulmonary vasodilation.

Differing from hypobaric hypoxia, marine mammals undergo routine breath-holding while diving and experience ischemia/reperfusion (I/R) events and hypoxemia (Allen and Vázquez-Medina, 2019). Northern elephant seals (*Mirounga angustirostris*), Weddell seals (*Leptonychotes weddellii*), bottlenose dolphins (*Tursiops truncatus*) and beluga whales (*Delphinapterus leucas*) all have blood CO levels that are higher than levels in healthy adult humans (Pearson et al., 2024; Pugh, 1959; Tift et al., 2014). Additionally, both elephant seals and bottlenose dolphins have extravascular CO concentrations in some tissues that are much higher than those found in healthy rodents and some tissues in humans (Piotrowski et al., 2021; Tift et al., 2023 preprint; Vreman et al., 2005, 2006). The high CO concentrations could be a result of elevated hemoprotein stores found in these species, and possibly safeguards these animals against developing injuries from the repeated hypoxia and I/R events that occur while diving (Tift and Ponganis, 2019).

If a modified HO–CO pathway is an evolutionary adaptation in high altitude and diving mammals, it is plausible that it could also be under evolutionary pressure in hypoxia-tolerant subterranean mammals. The African naked mole-rat (NMR) (*Hetercephalus glaber* Ruppell 1842) is a fossorial rodent species currently regarded as one of the most hypoxia-tolerant mammals (Buffenstein et al., 2022; Pamenter, 2022). They live in eusocial colonies of up to 300 individuals in burrows consisting of various chambers connected by tunnels (Brett, 1991). Considering the high number of animals gathering and respiring in such a confined space, it is likely that NMRs naturally experience variable bouts of hypoxia, with the most hypoxic conditions occurring in the most densely populated nest chambers (Brett, 1991; Buffenstein et al., 2022; Zions et al., 2020). Although the inevitability of NMRs experiencing periods of hypoxia in nature is debated, they have a notable array of physiological and behavioral adaptations that increase tolerance to hypoxia (Braude et al., 2021; Buffenstein et al., 2022). They exhibit a robust 50–70% decrease in both metabolic rate and ventilation while exposed to acute hypoxia and are capable of tolerating days to weeks at 8% O_2_, several hours at 3% O_2_ and 18 min in anoxia (Chung et al., 2016; Dzal et al., 2019; Pamenter, 2023; Pamenter et al., 2015, 2018; Park et al., 2017). Their response to hypoxia matches O_2_ supply to demand, preventing hyperventilation and is unique among mammalian responses to hypoxia (Dzal et al., 2019; Pamenter et al., 2014, 2015; Peng et al., 2022).

There is some evidence that NMRs have a modified HO–CO pathway that could assist in their hypoxia tolerance; however, the research is limited. For example, when compared with mice (*Mus musculus*), NMRs in normoxia have higher expression of *Hmox1* mRNA in liver tissue (Lewis et al., 2015). When exposed to hypoxia, CO production rates (indicative of HO activity) in NMR liver homogenates remain stable, whereas there is a drastic reduction in mouse liver homogenate CO production rates to almost undetectable levels after 1 h of hypoxia exposure (Peng et al., 2022). Similarly, the carotid body of NMRs has higher expression of *Hmox1* than mice in normoxia, but comparable expression of *Hmox2*. The mouse carotid body experiences reduced CO production by HO-2 during hypoxia, allowing hydrogen sulfide (H_2_S) production to occur, resulting in increased sensitivity to hypoxia. NMRs are known for their unique hypoxic ventilatory response and it has been suggested that higher HO-1 protein content in their carotid body helps to maintain CO production during hypoxia, suppressing the production of H_2_S that is necessary to increase the sensitivity of the carotid body to hypoxia (McCoubrey et al., 1997; Peng et al., 2022).

The objective of this study was to investigate the potential effects of acute and chronic hypoxia on CO concentration and overall HO activity in several tissues from NMRs (blood, brain, heart, intestine, kidney, liver, lung, ovaries or testes, hindlimb skeletal muscle and spleen). Our study also includes CO concentrations and HO activity in select tissues from normoxic mice for comparison to a similarly sized but hypoxia-intolerant rodent.

## MATERIALS AND METHODS

### Animals and ethics

African naked mole-rats (*Hetercephalus glaber* Ruppell 1842) were housed as colonies at University of Ottawa, Canada in interconnected rat and mouse cages using PVC tubing to mimic their natural burrow and tunnel habitats and were maintained at 30°C in 60% humidity with dim lighting on a 12 h:12 h light:dark cycle. The animals were provided with a diet of fresh fruits and vegetables and rodent chow and fed *ad libitum*. Animals were not fasted prior to hypoxia exposure. All experiments were approved by the University of Ottawa Animal Care Committee and conducted in accordance with the Animals for Research Act and by the Canadian Council on Animal Care (protocol #2535).

Mice (*Mus musculus* Linnaeus 1758) were housed at Beth Israel Deaconess Medical Center under normoxia (21% O_2_) in specific pathogen-free conditions with 12 h:12 h light:dark cycles. All procedures were approved by the Institution Animal Care and Use Committees at Beth Israel Deaconess Medical Center (protocol 106-2015 and 068-2015) before initiation and all procedures described herein conform to the committee's regulatory standards.

### Experimental design and sample collection

A total of 56 male and female nonbreeding (subordinate) adult NMRs were evenly and randomly divided into four treatment groups: (1) normoxia (control; 21% O_2_; *n*=14), (2) acute hypoxia for 4 h (7% O_2_; *n*=14), (3) acute hypoxia for 24 h (7% O_2_; *n*=14) or (4) chronic hypoxia for 7 days (11% O_2_; *n*=14). Of the 14 animals in each treatment group, tissues from selected individual animals were then utilized to measure CO concentration and/or HO activity (Table S1). NMRs exposed to 4 h of acute hypoxia were treated individually due to the time it takes for the experimental chambers to re-equilibrate after removing an animal. NMRs exposed to longer periods of hypoxia (24 h or 7 days) were treated in a colony grouping and animals were then sacrificed *en masse*. NMRs were not restrained in experimental chambers while being exposed to their designated treatments and were immediately and humanely euthanized after the exposure treatments were completed by acute conscious cervical dislocation followed by immediate decapitation. Male 8-week-old, CD-1 mice (*n*=12) were deeply anesthetized with isoflurane (3% v/v), and blood was sampled from the left ventricle after a thoracotomy. The heart was then transected for exsanguination. Whole blood was collected from both species and stored in glass capillary tubes coated in heparin and saponin to avoid coagulation. A stainless steel mixing bar was added to the glass tubes which were then capped with Hemato-seal tube sealing compound (Thermo Fisher Scientific, Waltham, MA, USA) on both ends (Vreman et al., 1984). Organs from mice and NMRs used to determine CO concentration and HO activity were immediately extracted upon euthanasia, blood rinsed from tissue with ice-cold saline, placed in cryovials and then immediately flash frozen in liquid N_2_. Samples collected from both species included: blood, brain, heart, intestine, kidney, liver, lung, hindlimb skeletal muscle (soleus and plantaris) and spleen. Ovaries or testes were also collected from NMRs.

### Measurement of endogenous carbon monoxide in extravascular tissues

Tissue CO extraction and quantitation was performed following previously established methods (Vreman et al., 2005, 1984). To extract CO from the collected organs, a section of frozen tissue (10–150 mg) was thawed and rinsed with chilled 0.1 mol l^−1^ potassium phosphate buffer (pH 7.4) to remove excess blood, placed into microcentrifuge tubes and then diluted 10-fold (weight/weight ratio) with chilled de-ionized water. The samples were diced using surgical scissors and then thoroughly homogenized using a tissue grinder (Ultra-Turrax T8, IKA Works, Inc., Wilmington, NC, USA) and Branson ultrasonic cell disruptor (SFX150, Brookfield, CT, USA). Using Hamilton gas-tight syringes attached to repeating dispensers, chilled tissue homogenate (10 µl) and 20% sulfosalicylic acid (20 µl) were injected into a glass amber chromatography vial (2 ml) with gas-tight septa that was previously purged with CO-free air (Reno, NV, USA). Contents were mixed thoroughly within the sealed vials and remained on ice for 15 min prior to analysis. The quantity of CO extracted from samples was determined using a reducing compound photometer gas chromatography system (Peak Performer 1, Peak Laboratories LLC, Mountain View, CA). Daily standard curves were generated prior to each experiment using a certified calibration gas (1.02 ppm CO balanced with nitrogen; Airgas, Radnor, PA, USA). Each sample was analyzed at least in duplicate. The CO concentrations in tissues were reported as pmol CO mg^−1^ wet weight (ww) tissue.

### Measurement of endogenous carbon monoxide and carboxyhemoglobin in blood

For whole blood samples, heparinized whole blood (1 µl) was injected into CO-free, purged chromatography vials (2 ml) with potassium ferricyanide (20 µl) using Hamilton gas-tight syringes (Vreman et al., 1984). The contents were mixed thoroughly and the vials remained on ice for 15 min prior to analysis. The CO values measured in the blood were reported as pmol CO mg^−1^ whole blood for comparison to the extravascular tissues (1 µl=approx. 1 mg), as well as *V*_CO_ (ml of CO dl^−1^ whole blood), which was used to determine COHb values using Eqn 1.

Concentrations of Hb (g dl^−1^) were determined using the cyanmethemoglobin method and Drabkin's Reagent (RICCA Chemical Co., Arlington, TX, USA). The maximum absorbance of cyanmethemoglobin at 540 nm was collected using a SpectraMax iD5 spectrophotometer (Molecular Devices, San Jose, CA, USA). A standard curve was generated daily with each experiment utilizing the Pointe Specific Hb standard (H7506STD, Canton, MI, USA).

The Hb concentration and *V*_CO_ values were used to calculate the percent of total Hb bound to CO (carboxyhemoglobin; COHb; % saturation) using the equation below, where the value of 1.34 is the Hüfner factor expressing the volume (ml) of CO that can bind to a gram of Hb (Ostrander et al., 1982; Vreman et al., 1984):
(1)

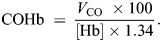


It has been recognized that CO can be generated from other processes (e.g. lipid peroxidation, photooxidation), which would contribute to the CO generation within the sample vials, resulting in inaccurate values (Vreman et al., 1990, 1998). Therefore, blank vials containing only CO extraction solutions used for extravascular tissue or blood CO quantification (i.e. sulfosalicylic acid or potassium ferricyanide, respectively), were purged at the same time as the sample vials and were used to correct for any background CO production that was not from the sample. These levels of CO were typically extremely low (∼1–2 pmol).

### Measurement of heme oxygenase activity

Maximum HO activity (i.e. combined HO-1 and HO-2 activity) in tissues was determined by quantifying the production of HO-generated CO via gas chromatography (Vreman and Stevenson, 1999). Frozen aliquots of organs (15–200 mg) were briefly thawed and rinsed with chilled 0.1 mol l^−1^ potassium phosphate buffer (pH 7.4) to remove any excess blood, placed into microcentrifuge tubes, and then diluted to 10–25% (weight/weight ratio) using 0.1 mol l^−1^ potassium phosphate buffer (pH=7.4). Samples were homogenized in the same methods used for CO-extracted samples. After being thoroughly homogenized, samples were centrifuged for 1 min at 13,000 ***g*** at 4°C and then the supernatant was transferred to a clean microcentrifuge tube. Using gas-tight syringes, 20 µl sample supernatant, 20 µl HO substrate (150 µmol l^−1^ heme and 15 µmol l^−1^ albumin) and 20 µl of 4.5 mmol l^−1^ NADPH (Thermo Fisher Scientific, Waltham, MA, USA) were added to sealed amber glass vials. Blank vials were also prepared for each sample to correct for any CO not produced from HO activity and contained 20 µl tissue supernatant, 20 µl HO substrate and 20 µl of 0.1 mol l^−1^ potassium phosphate buffer (pH 7.4). Sealed vials were incubated in a heated water bath for 5 min at 32°C, purged with CO-free air for 4–6 s, and then returned to the water bath to incubate for an additional 15 min. The incubation temperature was modified from the original method by Vreman and colleagues to account for the lower body temperature of NMRs (Buffenstein et al., 2022). Vials were then removed and placed on dry ice to terminate the HO reaction. All vials (samples and blanks) were analyzed at least in duplicate. Daily standard curves were generated prior to each experiment using a certified calibration gas (1.02 ppm).

Protein concentration in the tissue supernatant was determined using a Bradford Protein Assay (Bio-Rad, Hercules, CA, USA). A standard curve was generated with each experiment using bovine serum albumin (bioWORLD, Dublin, OH, USA). The absorbance (595 nm) of protein concentration was measured in triplicate readings using the SpectraMax iD5 spectrophotometer. Once the protein concentration of each sample was determined, HO activity was calculated by using the equation below and reported as nmol CO h^−1^ mg^−1^ protein:
(2)

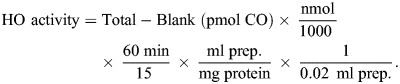


### Statistical analyses

All statistics were performed using JMP Pro 15.0.0 (SAS Institute Inc., Cary, NC, USA). Individual *n* values correspond to the number of individual animals within that data set (Table S1). Samples were analyzed in duplicate and the average of the measurements was used for statistical analyses. Written values are presented as means±s.d. Significant differences were determined by a *P*-value of ≤0.05. All data sets were tested for normality (Shapiro–Wilk test), homogeneity of variance (Levene test) and were transformed as needed to meet the assumptions of normal distribution. Non-parametric testing was used if data was unable to meet the assumptions.

An ANOVA was used to test for significant differences in both tissue CO concentration or overall HO activity between normoxic NMR organs, followed by a Tukey's honestly significant difference (HSD) *post hoc* test to determine the significant differences between the normoxic organs. Owing to much higher blood CO concentrations, when compared with extravascular tissues, blood was not included in the comparison analysis with extravascular tissues to avoid statistical biases. A separate ANOVA was then performed for each tissue type from NMRs (whole blood, brain, heart, intestine, kidney, liver, lung, reproductive, hindlimb skeletal muscle and spleen) to determine the effect of the hypoxia exposure treatments (4 h or 24 h at 7% O_2_ and 7 days at 11% O_2_) on response variables (tissue CO concentration, HO activity, *V*_CO_, Hb and COHb), in comparison to values from NMRs exposed to normoxia (21% O_2_). Dunnett's tests were used to determine significant differences in either tissue CO concentration or HO activity between hypoxic treatment groups and the normoxic group and Tukey's HSD tests were used to determine significant differences between the hypoxic treatment groups. The percentage change in tissue CO concentration and HO activity was calculated to compare the relative change in values from the different hypoxia exposures and normoxia (Fig. S1). Non-breeding NMRs do not undergo sexual maturation and lack circulating gonadal hormones; therefore, sex was not taken into consideration in our analyses (Holmes et al., 2009).

For the species comparison, separate ANOVAs were used to test for significant differences in tissue CO concentration (in all tissues except ovaries/testes) or overall HO activity (in heart, kidney, lung and spleen) between mice, normoxic NMRs and NMRs exposed to hypoxia treatments. Dunnett's tests, using mice values as the control, were used to determine significant differences in CO concentration or HO activity between mice and NMRs within a tissue.

## RESULTS

### Carbon monoxide concentrations in naked mole-rat extravascular tissues and blood

**Fig. 1.**
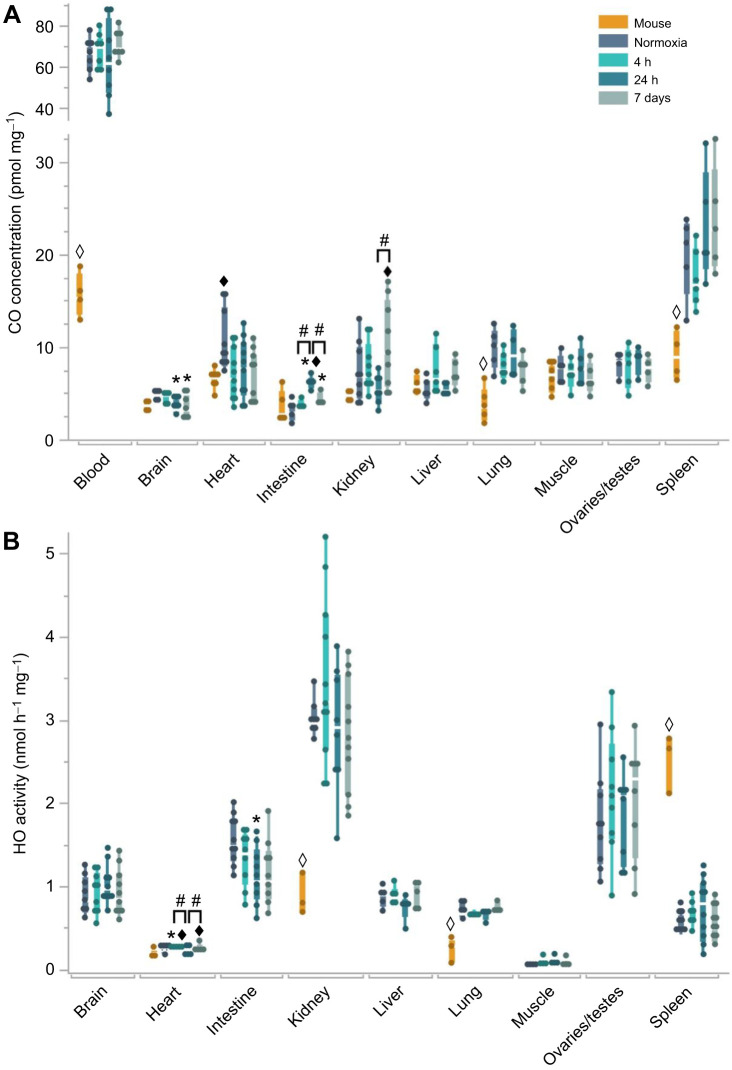
**Carbon monoxide (CO) concentration and heme oxygenase (HO) activity measured in naked mole-rats (NMRs) and mice.** (A) Concentration of CO (pmol CO mg^−1^ tissue) and (B) HO activity (nmol CO h^−1^ mg^−1^ protein) measured in NMRs exposed to normoxia (21% O_2_), acute (4 h or 24 h at 7% O_2_) or chronic hypoxia (7 days at 11% O_2_) and normoxic mice. Each data point represents the mean value for an individual animal based on duplicate values obtained from the same tissue. Sample sizes can be found in Table S1. *P≤0.05 hypoxic NMRs compared with normoxic NMRs. ^#^*P*≤0.05 between hypoxic NMR treatment groups (groups indicated by black bar). ^⧫^*P*≤0.05 NMR treatment group compared with mice. ^◊^*P*≤0.05 mice compared with all NMR groups.

**Fig. 2.**
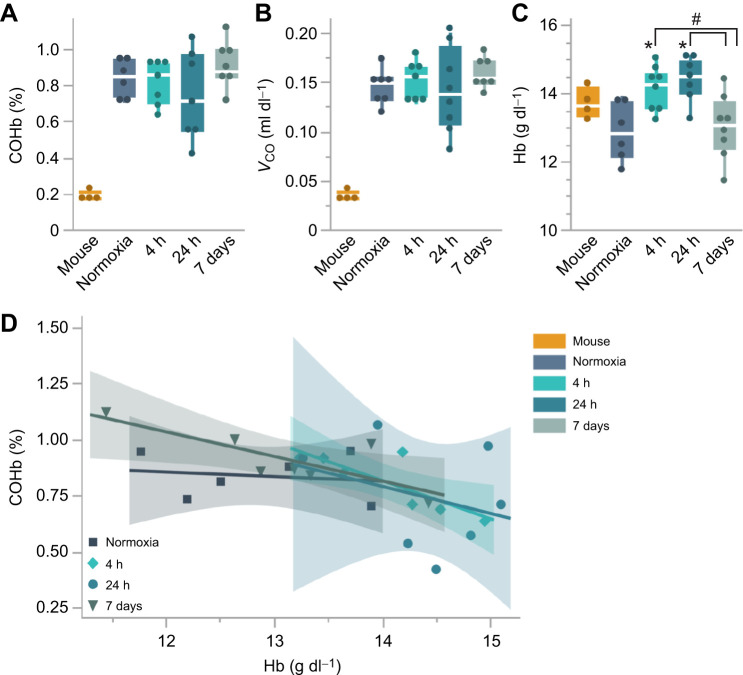
**Carboxyhemoglobin (COHb), CO bound to whole blood (*V*_CO_) and hemoglobin (Hb) concentration measured in NMRs and mice.** (A) COHb (% saturation), (B) *V*_CO_ (ml dl^−1^) and (C) Hb (g dl^−1^) in NMRs exposed to normoxia (21% O_2_), acute (4 h or 24 h at 7% O_2_) or chronic hypoxia (7 d at 11% O_2_) and normoxic mice. (D) Relationship between COHb with Hb in NMRs was only found to be significant after 4 h (*P*=0.02, *R*^2^=0.67, COHb_4h_=3.2–0.2*Hb) and 7 days (*P*=0.03, *R*^2^=0.63, COHb_7days_=2.4–0.1×Hb). All data for normoxia, 4 h, 24 h and 7 days represent values from NMRs and do not include mice. Each data point represents the mean value for an individual animal based on duplicate values obtained from the same tissue. Sample sizes can be found in Table S1. *P≤0.05 hypoxic NMRs compared with normoxic NMRs. ^#^*P*≤0.05 between hypoxic NMR treatment groups (groups indicated by black bar). ^◊^*P*≤0.05 mice compared with all NMR groups.

There was a significant difference in CO concentrations between the nine NMR organs exposed to normoxia (*F*_8,44_=18.2; *P*<0.0001). The highest mean extravascular tissue CO concentrations were found in the spleen (19.8±4.4 pmol mg^−1^), heart (10.4±3.4 pmol mg^−1^) and lung (9.8±2.2 pmol mg^−1^), while the lowest concentrations were measured in the intestine (2.8±0.8 pmol mg^−1^), brain (4.8±0.5 pmol mg^−1^) and liver (5.3±1.1 pmol mg^−1^) (Fig. 1A). The highest CO concentration was found in blood (65.1±8.0 pmol mg^−1^), which corresponds to a COHb value of 0.8±0.1% (Figs 1A and 2A).

Only the brain showed a significant decrease in CO concentration after exposure to hypoxia. When compared with normoxic NMRs (4.8±0.5 pmol mg^−1^), brain CO concentrations were significantly reduced after 24 h (3.5±0.7 pmol mg^−1^; *P*=0.03) and 7 days of hypoxia (3.5±1.1 pmol mg^−1^; *P*=0.03; Fig. 1A). Mean intestinal CO concentrations were elevated in all NMR treatment groups exposed to hypoxia when compared with normoxic values (2.8±0.8 pmol mg^−1^) but these increases only reached significance in animals exposed to hypoxia for 24 h (6.1±0.6 pmol mg^−1^; *P*<0.0001) or 7 days (4.4±0.7 pmol mg^−1^; *P=*0.01) (Fig. 1A and Fig. S1). Intestinal CO after 24 h of hypoxia was also significantly higher than after 4 h (3.8±0.5 pmol mg^−1^; *P*=0.0007) and 7 days of hypoxia (*P*=0.0007). NMR kidney CO concentration after 7 days of hypoxia was significantly higher than animals exposed to 24 h of hypoxia (10.3±4.6 pmol mg^−1^ and 5.4±1.7 pmol mg^−1^, respectively; *P*=0.03) but was not significantly higher than normoxic values (7.3±3.2 pmol mg^−1^) or levels in animals exposed to 4 h of hypoxia (7.8±2.6 pmol mg^−1^; Fig. 1A). There was not a significant change in CO concentrations in the heart, liver, lung, skeletal muscle, ovaries/testes or spleen from NMRs exposed to any treatment from this study.

None of the durations of hypoxia resulted in a significant difference in *V*_CO_ or COHb values (Fig. 2A,B). The mean Hb concentration in animals exposed to normoxia (12.9±0.9 g dl^−1^) was significantly lower than that in animals exposed to 4 h (14.1±0.6 g dl^−1^; *P*=0.03) or 24 h of hypoxia (14.4±1.0 g dl^−1^; *P*=0.007) (Fig. 2C). Concentrations of Hb after 4 h and 24 h were also significantly higher than after 7 days of hypoxia (13.0±0.9 g dl^−1^; *P*=0.04 and *P*=0.009, respectively); however, Hb concentration after 7 days of hypoxia was not significantly different to that in animals exposed to normoxia. Also, there was generally a negative relationship between NMR COHb and Hb concentration, but the relationship was only determined to be significant in blood from animals exposed to 4 h (*P*=0.02, *R*^2^=0.67) and 7 days of hypoxia (*P*=0.03, *R*^2^=0.63) (Fig. 2D).

### Comparison of carbon monoxide concentrations in extravascular tissues and blood from naked mole-rats and mice

There was a significant difference in CO concentrations in mice and at least one NMR treatment group in the heart, intestine, kidney, lung and spleen (Fig. 1A). Heart CO concentration in normoxic NMRs was significantly elevated compared with levels in mice (10.4±3.4 pmol mg^−1^ and 6.6±1.0 pmol mg^−1^, respectively; *P*=0.02). Intestinal CO was significantly elevated in NMRs after 24 h of hypoxia when compared with CO levels in mice (6.1±0.6 pmol mg^−1^ and 3.5±1.7 pmol mg^−1^, respectively; *P*=0.003). Kidney CO concentrations were significantly higher after 7 days of hypoxia in NMRs (10.3±4.6 pmol mg^−1^) when compared with mice (4.7±0.6 pmol mg^−1^; *P*=0.02). Concentrations of CO in mouse lung (3.7±1.9 pmol mg^−1^) and spleen (9.1±2.6 pmol mg^−1^) were both significantly lower than CO concentrations measured in all NMR treatment groups (*P*<0.05) (Fig. 1A).

The *V*_CO_ and COHb measurements were significantly lower in mice (0.03±0.01 ml dl^−1^ and 0.19±0.03%, respectively) when compared with NMR levels, regardless of hypoxia exposure (mean values for all treatments: 0.15±0.03 ml dl^−1^ and 0.83±0.17%, respectively, (*P*<0.05) (Fig. 2A,B). There was no significant difference in Hb concentrations between the two species, regardless of hypoxia treatments (Fig. 2C).

### Heme oxygenase activity in naked mole-rat extravascular tissues

There were significant differences in maximum, substrate-unlimited HO activity between the nine different organs tested in normoxic NMRs (*F*_8,55_=72.7; *P*<0.0001). The highest HO activities were found in the kidney (3.01±0.21 nmol h^−1^ mg^−1^), ovaries/testes (1.77±0.59 nmol h^−1^ mg^−1^) and intestine (1.54±0.29 nmol h^−1^ mg^−1^) (Fig. 1B). The lowest HO activities were found in the hindlimb skeletal muscle (0.06±0.02 nmol h^−1^ mg^−1^), heart (0.21±0.02 nmol h^−1^ mg^−1^) and spleen (0.60±0.11 nmol h^−1^ mg^−1^).

Only the NMR heart showed a significant increase in HO activity after exposure to hypoxia, with values being significantly higher after 4 h of hypoxia (0.27±0.02 nmol h^−1^ mg^−1^) when compared with normoxia (0.21±0.02 nmol h^−1^ mg^−1^; *P*=0.02). HO activity was significantly elevated in NMR hearts after both 4 h and 7 days of hypoxia (0.25±0.05 nmol h^−1^ mg^−1^) when compared with HO activity after 24 h (0.19±0.02 nmol h^−1^ mg^−1^; *P*=0.002 and *P*=0.02, respectively) (Fig. 1B). NMR intestinal HO activity was significantly decreased from normoxia levels (1.54±0.29 nmol h^−1^ mg^−1^) after 24 h of hypoxia (1.11±0.34 nmol h^−1^ mg^−1^; *P*=0.03). There was no significant change in HO activity in the brain, kidney, liver, lung, skeletal muscle, ovaries/testes or spleen from NMRs exposed to any treatment from this study.

### Comparison of heme oxygenase activity in extravascular tissues from naked mole-rats and mice

Maximal, substrate-unlimited HO activity was also measured in the heart, kidney, lung, and spleen of mice exposed to normoxia to compare to values from NMRs based on: (1) high CO concentration and/or HO activity in these NMR tissues, (2) changes in CO and/or HO activity in these NMR tissues after exposure to hypoxia, and (3) significant differences in CO concentrations in these tissues when NMRs were compared with mice. There was at least one treatment in NMRs that resulted in a significant difference in overall HO activity between mice and NMRs in each of the four tissues tested. The HO activity in mice hearts (0.18±0.05 nmol h^−1^ mg^−1^) was significantly lower than NMRs exposed to 4 h (0.27±0.02 nmol h^−1^ mg^−1^; *P*=0.004) and 7 days of hypoxia (0.25±0.05 nmol h^−1^ mg^−1^; *P*=0.02) (Fig. 1B). The mean HO activity for all treatments in NMR kidneys and lungs were significantly higher than mouse kidneys (0.88±0.25 nmol h^−1^ mg^−1^; *P*<0.0001) and lungs (0.24±0.14 nmol h^−1^ mg^−1^; *P*<0.0001). In contrast, mice had significantly higher HO activity in the spleen (2.51±0.35 nmol h^−1^ mg^−1^) when compared with all NMR treatments combined (0.62±0.24 nmol h^−1^ mg^−1^; *P*<0.0001) (Fig. 1B).

## DISCUSSION

This study highlights the different response of the HO–CO pathway in NMRs in relation to acute and chronic hypoxia, with major findings including: (1) NMRs have higher blood CO concentrations and COHb than mice from this study as well as mice from previous studies (Vreman et al., 2005, 2000a), (2) NMRs respond to acute hypoxia by transiently increasing Hb concentration; however, Hb concentration after 7 days of hypoxia is similar to values seen in animals only exposed to normoxia, (3) there is a negative relationship between NMR COHb and Hb concentration during certain durations of hypoxia, (4) NMRs in normoxia and hypoxia have higher CO concentrations in the lung and spleen when compared with mice, and (5) when compared with mice, NMRs in normoxia and hypoxia exhibit much higher HO activity in the kidney and lung, as well as much lower HO activity in the spleen.

A common response to hypoxia is to increase the red blood cells (RBCs) in circulation, in turn increasing Hb concentration and blood O_2_ carrying capacity. This can be achieved by a hypoxia-induced systemic increase in erythropoietin (EPO) levels, which upregulates erythropoiesis, or by splenic contraction, which releases stored RBCs and has been found in various conditions, such as diving and high altitude exposure (Espersen et al., 2002; Hurford et al., 1996, 1990; Reynafarje et al., 1959; Schagatay et al., 2020a,b; Sonmez et al., 2007; Stewart and McKenzie, 2002). For example, rainbow trout (*Oncorhynchus mykiss*) increase Hb by ∼15% after only 4 h of hypoxia (30% air saturation) (Lai et al., 2006). The Hb concentration remains elevated for up to 144 h of exposure to 30% air saturation and up to 216 h of exposure to 55% air saturation, and it was suggested that the initial increase was due to splenic contraction, while subsequent increases were likely related to erythropoiesis (Lai et al., 2006). Furthermore, rats exposed to chronic hypobaric hypoxia exhibit a continuous increase in Hb concentration for 3 weeks, not returning to baseline values for several weeks after the animals were brought back to normoxic conditions (Stutte et al., 1986). The NMRs in our study exhibited a significant increase in Hb concentration after 4 and 24 h of hypoxia, but values after 7 days were not different from those seen in animals exposed to normoxia, suggesting that the acute response may have been the result of splenic contraction rather than the production of newly circulating RBCs (Fig. 2C). A previous study of eight related African mole-rat species found unique, species-specific hematological responses to 3 h of acute hypoxia (5 kPa O₂). Only three species showed significant increases in hematocrit, and only the Highveld mole-rat (*Cryptomys hottentotus pretoriae*) showed a significant increase in hemoglobin concentration (Ivy et al., 2020). The Ansell's mole-rat (*Fukomys anselli*) was found to downregulate erythropoiesis after chronic intermittent hypoxia and decrease RBCs, hematocrit and Hb concentrations, but it should be noted that O_2_ measurements within the habitat did not fall below 10% (Henning et al., 2024). It is possible that NMRs would not need to rely on increased O_2_-transport capacity from erythropoiesis during hypoxia, as it has been suggested that metabolically suppressed NMRs have the ability to survive at 11% O_2_ without utilizing the many O_2_-saving mechanisms they have available (Farhat et al., 2020). Further investigation is needed to determine whether increased Hb in NMRs is a response to the severity of hypoxia (7% versus 11% O_2_) or the duration of hypoxia (4 h and 24 h versus 7 days).

Despite changes in Hb concentration, we found that overall *V*_CO_ and COHb levels in NMRs were not significantly altered by hypoxia exposure (Fig. 2A,B). If the elevated Hb was a response to acute hypoxia, it is possible increased RBC destruction occurred after 24 h of hypoxia exposure, but the additional CO produced may have been removed before day 7 and was undetected because of our experimental design. The elevated NMR blood CO concentrations and COHb, regardless of exposure conditions, compared with the mice in this study, could possibly be the result of less CO being removed from the NMR body over time, owing to reduced hearts rates and respiratory rates compared with mice (256 beats min^−1^ and <1 breaths s^−1^ in NMRs versus 704 beats min^−1^ and ∼3 breaths s^−1^ in mice) (Carraway et al., 2000; Grimes et al., 2014; Park et al., 2017; Vreman et al., 2005). These rates are reduced even further in NMRs when exposed to hypoxia, with exposure to 9% O_2_ for 1 h resulting in decreases in respiratory rate (∼25%) and heart rate (17%) (Pamenter et al., 2019).

Our findings also show that NMRs have the opposite relationship to that of elephant seals and humans at high altitude regarding COHb and Hb concentrations (Fig. 2D). Adult Peruvians (4330 m) exhibit a positive relationship between COHb and Hb, with the same relationship seen in elephant seals, possibly indicating increased RBC destruction, higher HO activity in tissues, or reduced CO removal due to breath-holding and sleep apnea (Tift et al., 2020, 2014). The reversed relationship between COHb and Hb in NMRs could indicate shifts in the rates of CO production and removal that differ from other hypoxia-tolerant species and populations. It should be noted, however, that the magnitude of change in COHb with Hb concentrations was very low in NMRs when compared with those seen in elephant seals and Peruvians.

Initially, an upregulation in the cerebral HO–CO pathway in NMRs exposed to hypoxia might be expected based on previous connections found in other species between the upregulation of *Hmox2* and CO production to brain homeostasis and neuronal survival during hypoxia (Mahan, 2012; Parfenova and Leffler, 2008). One of the potential consequences of cerebral hypoxia is excitotoxic levels of extracellular glutamate, an important neurotransmitter that has been shown to stimulate CO production via HO-2 activity (Kanu and Leffler, 2007; Meldrum, 2000; Robinson et al., 2002). HO-2 is found in abundance in the mammalian brain and previous findings have suggested that, with few exceptions, it is predominately cerebral HO-2-generated CO that is rapidly upregulated during acute hypoxia and responsible for the vasodilatory effects in the brain (Kanu and Leffler, 2007; Maines, 1997; Parfenova and Leffler, 2008). NMRs exposed to hypoxia, however, have been found to decrease cerebral glutamate levels, potentially explaining our finding of decreased cerebral CO concentrations with time spent in hypoxia (Fig. 1A) (Cheng et al., 2022). Additionally, although NMRs exposed to 24 h of hypoxia (7% O_2_) have increased lipid peroxidation in the liver, kidney and skeletal muscle, there is a reduction in brain lipid peroxidation (Hadj-Moussa et al., 2022). Considering that lipid peroxidation is a potential source of endogenous CO production, the lack of glutamate to induce HO-2 activity, as well as the decreased CO production via lipid peroxidation in the brain could explain the decrease in the overall CO concentration in this organ over time in hypoxia (Vreman et al., 1998; Wolff and Bidlack, 1976).

As mentioned previously, NMRs are capable of significantly reducing their metabolic rates during hypoxia exposure, to include significant cerebral metabolic suppression (Buffenstein et al., 2022; Edrey et al., 2011; Farhat et al., 2021, 2020; Pamenter, 2022; Pamenter et al., 2018). When exposed to hypoxia (7% O_2_) for 24 h, NMRs decreased gene and protein expression of two ATP-dependent heat shock proteins (HSPs; HSP27 and HSP40), as well as decreasing gene expression of ATP-independent *Hsp70* and *Hsp90*, suggesting that cerebral energy conservation is prioritized over cytoprotective protein chaperoning (Nguyen et al., 2019). Furthermore, NMRs exhibited reduced cerebral glycolytic activity after 1 h of hypoxia (3% O_2_) (Ojaghi and Pamenter, 2025). After 4 h of hypoxia exposure (3% O_2_), the NMR brain suppressed energy-expensive processes, such as *de novo* protein translation, cellular proliferation and apoptosis while activating a cerebral HIF-1α-centered response (Hadj-Moussa et al., 2021; Hawkins et al., 2019). Since the NMR brain exhibits a greater reliance on metabolic suppression rather than on traditional neuroprotective strategies such as enhanced anaerobic glycolysis or increased antioxidant enzyme expression, this may help to explain the decrease in brain CO concentration and why HO activity did not change.

Regardless of the decrease in brain CO concentration over time in hypoxia, the absolute values are relatively low when compared with other organs tested (Fig. 1A). This is unsurprising and the trend of low CO in cerebral tissue has also been found in bottlenose dolphins, healthy rodents and deceased humans (Tift et al., 2023 preprint; Vreman et al., 2005, 2006). Low cerebral CO has generally been attributed to the low solubility of the gas in lipids, which are at high concentration in neural tissues, as well as due to a low abundance of CO-binding heme proteins when compared with other organs (Vreman et al., 2006).

Gastrointestinal disruptions are common as a result of exposure to hypoxia and are primary symptoms of acute mountain sickness (Netzer et al., 2013; Yu et al., 2016). HO-1 was found to be upregulated in mesenteric arteries from rats exposed to chronic hypoxia, resulting in smooth muscle hyperpolarization and vasodilation (Naik et al., 2003). However, we found a general trend of decreased maximal intestinal HO activity over time in hypoxia and a slight increase in intestinal CO concentration over time in response to hypoxia exposure (Fig. 1A,B). This could be attributed to the unique gut microbiome found in NMRs. It has been recognized that the gut microbiome is capable of producing and consuming CO, and some bacteria possess HO-like enzymes with the ability to produce CO (Hopper et al., 2020; Maharshak et al., 2015; Tomasova et al., 2016). Multiple studies have identified the NMR microbiota to include several microbial species that have the capability to produce CO (Cong et al., 2018; Debebe et al., 2017, 2016; Hopper et al., 2020; Vreman et al., 2000a). Interestingly, the NMR microbiota also includes bacteria of the family *Desulfarculaceae*, which can oxidize CO to CO_2_ and had previously never been detected within an animal microbiota, which in turn could be masking further increases in intestinal CO production (Debebe et al., 2017; Kuever, 2014; Rabus et al., 2015)*.* Further studies investigating the impacts of the NMR gut microbiota on CO production and oxidation rates are needed to accurately detect the response in the HO–CO pathway during hypoxia.

Previous studies have reported that the cardiopulmonary HO–CO pathway is altered during hypoxia in various species, but the responses are inconsistent. Increased HO and CO in the heart reduce damage from I/R injuries and counteract cardiac dysfunction caused by chronic heart failure. Increased HO and CO in the lungs not only prevent hypoxia-induced pulmonary arterial hypertension (PAH) but also reverse established PAH and reduce right heart mass (Herrera et al., 2008; Otterbein et al., 2016; Yet et al., 2001; Zuckerbraun et al., 2006). Increased expression of HIF-1α in cardiac tissue of NMRs exposed to normoxia is met with a down-regulation of downstream hypoxia responsive genes (Faulkes et al., 2024). Considering that HO-1 is highly regulated by HIF-1α, this could help to explain why HO activity in NMRs during hypoxia was significantly higher than mice from this study.

The cardiac HO–CO pathway response to hypoxia is also influenced by the lung and pulmonary circulation. Increased *Hmox1* expression has been found in rat cardiomyocytes exposed to hypoxia as a response to pressure overload of the right ventricle, an indication of hypoxic pulmonary vasoconstriction (HPV) (Katayose et al., 1993). Rats exposed to hypobaric hypoxia exhibit a threefold increase in HO activity in the lungs after 24 h of hypoxia, followed by suppression of HO activity back to baseline values from days 3–21 of hypoxia that coincides with the development of polycythemia, right ventricular hypertrophy and pulmonary remodeling (Carraway et al., 2000). Based on the previously reported upregulated HO–CO pathway in neonatal llamas at high altitude, we expected an increase in lung CO concentration and HO activity in NMRs exposed to hypoxia as an adaptation to prevent HPV (Herrera et al., 2008). Differing from these previous studies, NMRs maintained relatively low HO activity in the lung when compared with other organs (Fig. 1B). As the lung is the primary location of CO excretion from the body, the higher blood CO concentration and COHb in NMRs, when compared with mice and rats, could help to explain the higher lung CO concentration in NMRs (Figs 1A and 2A) (Vreman et al., 2005).

An increasing number of studies have identified the HO–CO pathway as having a vital role in regulating renal function and renal medullary blood flow, which has a role in long-term control of arterial blood pressure (Cowley, 1992; Cowley et al., 1995; Zou et al., 2000). HO-2 protein is found in abundance in the kidney and overall HO activity increases in the kidneys as a response to higher circulating Hb (Abraham et al., 2009; Ryter and Choi, 2006; Vreman et al., 2000a). We show that NMRs exhibit an increase in Hb concentration during acute hypoxia (Fig. 2C), although we did not see an upregulation in kidney HO activity with time spent in hypoxia (Fig. 1B). However, NMRs did have much higher kidney HO activity (mean values for all treatments; 3.05±0.3 nmol h^−1^ mg^−1^) when compared with the mice in this study, as well as newborn rhesus monkeys and rats (0.88±0.25 nmol h^−1^ mg^−1^, 0.12±0.07 nmol h^−1^ mg^−1^ and 1.32±0.54 nmol h^−1^ mg^−1^, respectively) (Vreman and Stevenson, 1988, 1999).

As mentioned previously, hypoxia can induce an increase in EPO levels, which is produced in the kidneys. Also, EPO has a stimulatory effect on HO-1 expression in the kidney that is believed to have cytoprotective effects against oxidative stress (Katavetin et al., 2007). When blind mole-rats (*Spalax* spp.) and rats were exposed to chronic hypoxia, EPO gene expression in *Spalax* kidney increases over the first 24 h of hypoxia and is elevated when compared with levels in rats (Shams et al., 2004). The same study also found *Spalax* kidney has higher hypoxia-inducible factor 1α (HIF-1α) gene expression than that in rats during the first 24 h of hypoxia. HIF not only regulates EPO expression but also increases HO expression (Lee et al., 1997; Yang et al., 2003). Although the EPO response to hypoxia has not been investigated in NMRs, they do have higher HIF-1α expression in the kidney during normoxia and increased HIF-1α expression in the kidney after 15 h of hypoxia compared with levels in mice (Xiao et al., 2017). Further studies including the EPO response to hypoxia in NMRs may provide more insight into their elevated maximum HO activity in the kidneys.

The spleen and liver are recognized as primary sites for RBC and Hb degradation and are generally thought to have higher CO concentrations and HO activity when compared with other organs, although that has not consistently been found across studies (Tenhunen et al., 1968; Tift et al., 2023 preprint; Vreman et al., 2005). Although the spleen did have the highest CO concentration when compared with the other organs, the NMRs in our study did not exhibit a statistically significant change in CO concentration or maximum HO activity in either the spleen or the liver after exposure to acute and chronic hypoxia (Fig. 1A,B). Our findings in the liver were not unexpected since a previous study found that exposure to hypoxia for 1 h does not alter CO production in NMR livers, whereas there is a decrease in CO production in mouse livers after hypoxia exposure (Peng et al., 2022). Exposure to hypobaric hypoxia also reduces splenic HO-1 protein in mice and inhibits erythrophagocytosis, RBC clearance and iron recycling, which results in a buildup of RBCs within the spleen (Yang et al., 2023). NMRs may maintain low HO activity in both normoxic and hypoxic environments as a way to ensure necessary RBC clearance and iron recycling for uninterrupted RBC production, as well as to prevent potential hypoxia-induced polycythemia.

Our study found normoxic mice to have significantly higher maximum splenic HO activity than NMRs exposed to normoxia or hypoxia (Fig. 1B). The generally low HO activity in the NMR spleen may be attributed to their unique reliance on the spleen as a major site for erythropoiesis. In most adult mammals, erythropoiesis occurs predominately in the bone marrow and the spleen is primarily utilized to recycle aged RBCs; however, NMRs produce RBCs in both the bone marrow and spleen during basal conditions, with the spleen as the primary site of erythropoiesis (Emmrich et al., 2022; Mebius and Kraal, 2005). The overall maintenance of low HO activity in the spleen and liver also indicates the possibility of low RBC turnover rates, linked to a long RBC lifespan. A previous study suggested that *Hmox1* deficiency in mice increases RBC lifespan, potentially by attenuating macrophage-mediated RBC removal in the spleen, as well as the liver and bone marrow (Fraser et al., 2015). Although RBC lifespan has not been measured in NMRs, they have larger RBCs, elevated mean corpuscular volume (MCV) and higher Hb and hematocrit when compared with mice (Bégay et al., 2022; Emmrich et al., 2022). Higher MCV has also been connected to a longer RBC lifespan and this should be investigated further in future studies (Aldrich et al., 2006).

The HO pathway is vital to skeletal muscle as it is the only mechanism that clears released heme and is necessary for maintaining tissue damage control and muscle health (Alves de Souza et al., 2021). Skeletal muscle in mice, as well as in bottlenose dolphins and northern elephant seals, has been identified as one of the organs to have high tissue CO concentrations relative to other organs (Piotrowski et al., 2021; Tift et al., 2023 preprint; Vreman et al., 2005). Increased CO concentrations in skeletal muscle are speculated as being the result of either higher myoglobin content acting as a potential source for CO production or sink for endogenously produced CO via HO activity. Although there is limited information on myoglobin in NMRs, elevated myoglobin has been reported in other fossorial rodents (e.g. *Spalax*) when compared with rats (Avivi et al., 2010; Widmer et al., 1997). The low HO activity in NMR skeletal muscle suggests there is low heme turnover in the tissue, regardless of O_2_ availability. Although only conjecture, hypoxia alone might not induce HO activity in the NMR skeletal muscle, but may require additional stimuli (e.g. ischemia) (Bergeron et al., 1997; Vesely et al., 1998).

High *Hmox2* expression is found in the testes of other mammals and research has demonstrated protective effects of HO-1 in testicular I/R injuries in rodents (Maines, 1997; Reyes et al., 2012; Xie et al., 2024; Yang et al., 2007). There is also evidence that HO has a role in female reproductive physiology, with HO-1 defining ovulation and the changes in hormones throughout the estrous cycle influencing HO-1 protein concentrations in rodents (Cella et al., 2006; Kreiser et al., 2003; Odrcich et al., 1998; Zenclussen et al., 2014, 2012). However, NMRs are eusocial mammals known to have a queen that breeds with one to three select males while the remainder of the colony is sexually suppressed and do not undergo sexual development (Faulkes and Abbott, 1991; Faulkes et al., 1991; Holmes et al., 2009). While remaining in a colony with a breeding female, non-breeding females have ovaries that are pre-pubescent in appearance and there is the absence of a follicular cycle and ovulation (Faulkes et al., 1990; Kayanja and Jarvis, 1971; Westlin et al., 1994). Despite knowing that HO is used in the ovaries during estrous cycles, there is little information about the specific relationship between the HO–CO pathway and the NMR and their estrous cycle. Additionally, there is still little known about the response to hypoxia in reproductive NMRs and in general, and our understanding of the effects of hypoxia on the HO–CO pathway in the female reproductive system is relatively limited and requires further investigation (Němeček et al., 2017; Ojaghi and Pamenter, 2024, 2025).

The overall absence of significant changes in the HO–CO pathway in NMRs exposed to hypoxia may not reflect the absence of sensitivity to hypoxia severity but may instead indicate an adapted habituated tolerance to hypoxia. Given their extreme hypoxia tolerance, the baseline physiology of NMRs may already be optimized for an environment with decreased O_2_ availability, limiting the need for compensatory responses to chronic hypoxia. While NMRs exhibited metabolic plasticity after exposure to 8–10 days of chronically sustained hypoxia (8% O_2_), they did not exhibit ventilatory plasticity (Chung et al., 2016). In addition, it has been demonstrated that the glutamatergic signaling involved in ventilatory and metabolic regulation in normoxia are not involved in the NMR ventilatory response to either acute or chronic hypoxia (Dzal et al., 2019). This could potentially protect their tissues from their subterranean environment that frequently experiences variable levels of hypoxia. It was further shown that NMRs exposed to 4 weeks of chronic hypoxia (11% O_2_) exhibit suppression of brain metabolic enzymes and reduced Na^+^/K^+^-ATPase activity, reflective of an energy-conserving, hypometabolic state rather than a stress-induced physiological response associated with high energetic cost (Farhat et al., 2020). Therefore, it is possible that the lack of significant change in tissue CO and/or HO activity with hypoxia exposure in the majority of tissues tested may be an indication of hypoxia habituation rather than an absence of a response.

It is not unexpected that values of maximum HO activity, measured in extravascular tissues from this study, do not always reflect CO concentrations in the same tissue. This is because the maximum substrate-unlimited HO activity measured in this study probably does not reflect the actual *in vivo* HO activity in the tissue at the time of tissue collection. In contrast, CO concentrations in the extravascular tissues do reflect the amount of CO in that tissue at the time of collection. For example, HO activity from healthy rat liver and heart was reported as 0.57±0.12 and 0.10±0.07 nmol h^−1^ mg^−1^ protein, respectively (Vreman et al., 2000b). Meanwhile, CO concentrations from healthy rat liver and heart tissue were reported as 4±1 pmol mg^−1^ and 6±3 pmol mg^−1^, respectively (Vreman et al., 2005). In addition, very few studies have simultaneously measured extravascular CO concentrations and heme oxygenase activity in multiple tissues from animals, making comparisons of the two values difficult between species. Moreover, most studies that have investigated HO activity in tissues measure bilirubin production over a period of time, as opposed to CO produced over time. Considering that CO is the direct product of HO activity, we strongly suggest this be adopted as the gold-standard for HO activity measurements in tissues, especially considering that bilirubin production requires biliverdin to be produced first from HO activity, followed by the conversion of biliverdin to bilirubin by biliverdin reductase. Bilirubin production in a tissue over time therefore considers the activity of two enzymes, which cannot assume to be directly correlated in their activity without proper justification.

Although there are a few studies that evaluate HO-1 and/or HO-2 gene or protein expression in this species, they either do not explore both acute and chronic hypoxia or they offer limited tissues analyses (Edrey et al., 2014; Lewis et al., 2015; Peng et al., 2022). Exploring how HO-1 and HO-2 expression and associated enzymatic activity are affected in different tissues during acute and chronic hypoxia, and by varying severities of hypoxia, would provide valuable insight into how the HO–CO pathway responds to hypoxia. It is safe to assume that NMRs experience intermittent hypoxia in the wild and undergo periods of hypoxia-reoxygenation (Buffenstein et al., 2022). Designing experiments to evaluate the effects of intermittent hypoxia as well as reoxygenation on the HO–CO pathway after hypoxia may provide insight into how these enzymes are functioning under normal physiologic conditions in the wild. Additional pathways of CO production not directly linked to HO activity should also be further investigated (Debebe et al., 2017; Kuever, 2014; Rabus et al., 2015)*.*

Both euthanasia and tissue collection protocols for NMRs and mice for this study ensured that tissues were harvested, rinsed free of blood and flash frozen as quickly as possible in order to maximize quality of the tissue for analyses. Owing to the majority of CO in tissues being bound to hemoproteins, the high affinity of hemoproteins for CO, and the minimization of time organs spent exposed to ambient air, we believe the CO concentrations in tissues from this study accurately reflect *in vivo* values (Vreman et al., 2001). Moreover, our methods to measure extravascular tissue CO in mice reflect similar values previously reported in the literature (Vreman et al., 2005).

In conclusion, our study provides baseline information for tissue CO concentrations and overall HO activity in nine different organs during normoxia and both acute and chronic hypoxia exposure in NMRs. While there are limited studies available, hypoxia-intolerant species have reduced or impaired HO–CO responses to chronic hypoxia, while hypoxia-tolerant species have modified HO–CO pathways. Our findings align with these previous studies and show that the hypoxia-tolerant NMR can maintain HO–CO activity during both acute and chronic hypoxia in most organs tested. NMRs also generally express either similar or elevated tissue CO and HO activity in most of the organs tested when compared with mice, with the exception of splenic HO activity. Although there is still much to be learned, our study suggests that the HO–CO pathway may have evolved similarly to high altitude and marine species in a hypoxia-tolerant and fossorial species. Understanding how this pathway has evolved in the NMR, as well as other hypoxia-tolerant species, may provide valuable insight into treating injuries associated with hypoxia.

## Supplementary Material

10.1242/jexbio.250502_sup1 Supplementary information
