# Supplementary material for: Impact of acute and chronic hypoxia on the heme oxygenase/carbon monoxide pathway in naked mole-rats (Heterocephalus glaber)
Source: J Exp Biol. 2025 Dec 12;228(24):jeb250502. doi: 10.1242/jeb.250502 (PMC12752505; doi:10.1242/jeb.250502)
Supplement: Supplementary information [file jexbio-228-250502-s1.pdf]

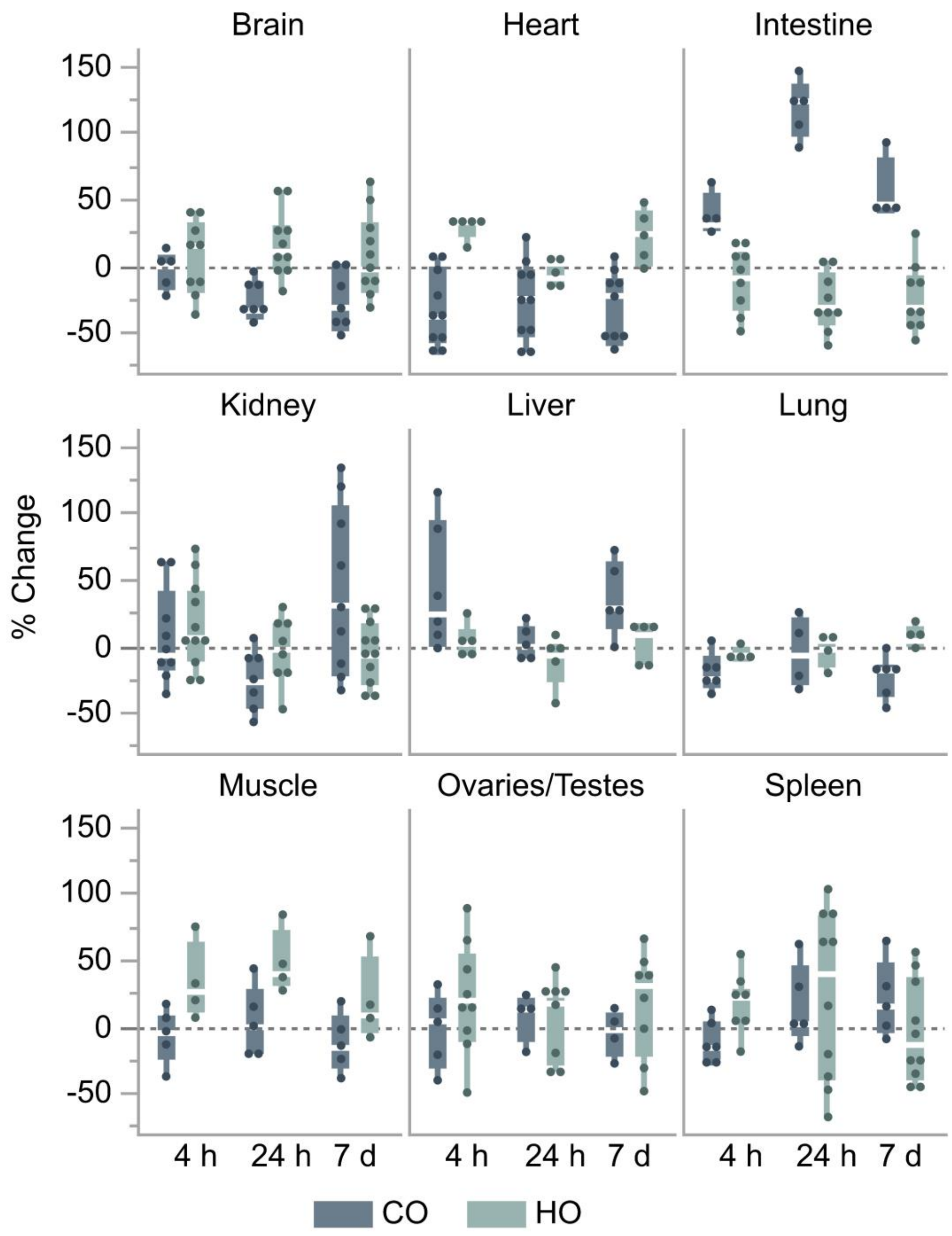

Fig. S1. Percent change in CO concentration and HO activity in NMRs after hypoxia exposure. Percent change from mean normoxic values in CO concentrations and HO activity in NMRs exposed to either acute (4 h or 24 h at 7% O_2) or chronic hypoxia (7 d at 11% O_2). Each data point represents the mean value for an individual animal based on duplicate values obtained from the same tissue. Sample sizes can be found in Table S1.

Table S1. Number (*n*) of individual naked mole-rats and mice used to determine tissue-specific carbon monoxide (CO) concentrations and heme oxygenase (HO) activity. Dashes in the table signify that mice samples were not tested for CO concentration and/or HO activity for the respective organ.

	Naked Mole-Rats				Mice
	Normoxia	4 h	24 h	7 d	Normoxia
**Blood**					
CO	7	7	8	7	4
**Brain**					
CO	5	5	7	7	4
HO	8	9	10	10	-
**Heart**					
CO	10	10	10	9	8
HO	5	5	5	5	3
**Intestine**					
CO	5	4	5	4	5
HO	10	9	9	9	-
**Kidney**					
CO	9	9	7	9	4
HO	8	11	8	11	3
**Liver**					
CO	5	6	5	5	4
HO	5	5	5	5	-
**Lung**					
CO	5	6	4	6	5
HO	5	4	4	4	3
**Muscle**					
CO	5	5	5	5	7
HO	4	4	4	4	-
**Ovaries/Testes**					
CO	4	5	4	4	-
HO	9	9	8	8	-
**Spleen**					
CO	5	6	5	5	4
HO	9	7	10	10	3

Journal of Experimental Biology • Supplementary information
